# Supplementary material for: Content-rich biological network constructed by mining PubMed abstracts
Source: BMC Bioinformatics. 2004 Oct 8;5:147. doi: 10.1186/1471-2105-5-147 (PMC528731; doi:10.1186/1471-2105-5-147)
Supplement: Additional File 2 — The original results of the above study (non-essential files are deleted to keep the file size under the limit set by BMC bioinformatics). [file 1471-2105-5-147-S2.bz2 › chilibotAdditionalFile2/dip05/583ID9533885E10951/html/NUTF2_RAN.html]

 


 **NUTF2** and **RAN** 
  
Found 47 abstracts in PubMed, retrieved 05.  
 

 What does Google say? 
 PDF only 
| .edu only 

---

**Interactive relationship** (e.g. stimulation, inhibition, etc)

**Inhibitory relationship**- Engineered mutants at the interaction interface reduce the binding of NTF2  [ **NUTF2** ]  to nuclear pores and cause reduced growth rates and  **Ran**  mislocalization when substituted for the wild type protein in yeast.  Ref: 12065398 EMBO J, 2002
**Neutral relationship**- NTF2  [ **NUTF2** ]  mediated import of  **Ran**  appears to be the bottleneck for maximal capacity of  **Ran**  driven nuclear transport.  Ref: 12606574 EMBO J, 2003

**Non-interactive relationship** (e.g. studied together, co-existance, homology, etc.)

- In contrast, a significant reduction in affinity of the RanGDP binding protein NTF2  [ **NUTF2** ]  for  **Ran**  CoreGDP is observed, indicating that the switch regions have changed conformation in the  **Ran**  Core mutant.  Ref: 12051861 J Mol Biol, 2002
- Four mutant complementation groups were characterized that correspond to respective mutations in genes encoding  **Ran**  gsp1, and essential  **Ran**  regulatory factors  **Ran**  GTPase  [ **RAN** ]  activating protein rna1,  **Ran**  guanine nucleotide exchange factor prp20, and the RanGDP import factor ntf2  [ **NUTF2** ] .  Ref: 12654904 J Cell Biol, 2003
